# High-density genetic linkage-map construction of hawthorn and QTL mapping for important fruit traits

**DOI:** 10.1371/journal.pone.0229020

**Published:** 2020-02-11

**Authors:** Yuhui Zhao, Yidi Zhao, Yinshan Guo, Kai Su, Xiaochang Shi, Di Liu, Jijun Zhang

**Affiliations:** 1 College of Horticulture, Shenyang Agricultural University, Shenyang, P.R.C; 2 National and Local Joint Engineering Research Center of Northern Horticultural Facilities Design and Application Technology, Shenyang, P.R.C; 3 College of Horticulture Science and Technology, Hebei Normal University of Science and technology, Qinhuangdao, P.R.C; North Dakota State University, UNITED STATES

## Abstract

Few reports exist on QTL mapping of the important economic traits of hawthorn. We hybridized the cultivars ‘Shandongdamianqiu’ (female parent) and ‘Xinbinruanzi’ (male parent), and 130 F_1_ individuals and the two parents were used for RAD-seq, SNP development, and high-density linkage map construction. Three genetic maps were obtained, one for each of the parents and an integrated one. In these three maps, 17 linkage groups were constructed. The female and male parent maps contained 2657 and 4088 SNP markers, respectively, and had genetic distances of 2689.65 and 2558.41 cM, respectively, whereas the integrated map was 2470.02 cM, and contained 6,384 SNP markers. QTL mapping based on six agronomic traits, namely fruit transverse diameter, vertical diameter, single fruit weight, pericarp brittleness, pericarp puncture hardness, and average sarcocarp firmness were conducted, and 25 QTLs were detected in seven linkage groups. Explained phenotypic variation rate ranged from 17.7% to 35%. This genetic map contains the largest number of molecular markers ever obtained from hawthorn and will provide an important future reference for fine QTL mapping of economic traits and molecular assisted selection of hawthorn.

## Introduction

Hawthorn (*Crataegus spp*., 2n = 2X = 34), belongs to the *Rosaceae* family and is mainly distributed in North America, Europe, Asia and other temperate regions of the northern hemisphere[1]. China is one of the centers of origin of hawthorn berry-trees, and the species are widely cultivated in the provinces of ‘Shandong,’ ‘Shanxi,’ ‘Liaoning,’ and ‘Hebei’. Hawthorn is a typical edible and medicinal plant [2–3] with important value both as table fruit and in processing. Hawthorn berries contain a variety of essential nutrients and micronutrients; to date, more than 150 distinct compounds have been detected, which is of great value for product development and research [4–8]. As an important index of fruit quality, fruit appearance and texture directly affect the nature of the fruit as a market commodity and its storability.

Like other perennial woody fruit-trees, hawthorn has a long growth cycle and a complex genetic background; most agronomic traits related to yield, dimensions, nutrition quality, storage ability, and disease resistance are quantitative traits, i.e., they are controlled by multiple genes. Traditional breeding strategies usually require a long time and breeding efficiency is poor. Therefore, QTL-mapping based on molecular genetic mapping can provide an important reference for marker-assisted selection of important economic traits of hawthorn, whereby breeding cycles could be significantly shortened[9].

Single nucleotide polymorphism (SNP) markers are third-generation molecular markers that refer to sequence polymorphisms caused by the variation of a single nucleotide on the genomic sequence. SNP markers are abundant and widely distributed in most species, with good genetic stability and high accuracy. In recent years, the development of next generation sequencing technology has greatly promoted the development of SNP markers for wide use in genetic diversity and evolutionary analysis, genome association analysis, high-density genetic map construction and QTL mapping of many species [10–14]. RAD sequencing was one of the major strategies to develop SNP markers. It was a technology of reduced-representation genome sequencing (RRGS), with the advantages of simple operation, low experimental cost, high throughput and independent of any genome information [15–17]. Until now, RAD sequencing has been applied to many species. Pfender et al.[15] conducted RAD-seq based on two parents and 191 of their hybrid offspring and then constructed a linkage map. Finally, three QTLs related to ryegrass rust disease were discovered. Wang et al. [18] constructed a high density linkage map that contained 1522 SNP markers for sesame based on RAD-seq, and discovered 50 QTLs related to sesame stem length and seed coat color. In recent years, many researchers of fruit trees have also reported genetic map construction based on RAD sequencing technology. Sun et al.[19] constructed the linkage maps of apple cultivar ‘Jonathan’ and ‘Golden Delicious’ based on RAD-seq, containing 2017 and 1932 SNP markers, respectively, and discovered 12 QTLs related to fruit weight, fruit firmness, sugar content and fruit acidity; Similarly, Wu et al.[20] constructed a high density linkage map for pear by using RAD-seq. The integrated map contained 3143 SNP markers, which were then were used for QTL mapping of 11 important agronomic traits of pear, namely length of pedicel, single fruit weight (SFW), soluble solid content, transverse diameter (TD), vertical diameter (VD), calyx status, flesh color, juice content, number of seeds, skin color, and skin smooth. Further, Zhao et al.[21] constructed a high-density linkage map that contained 2748 SNP markers for jujube, and Zhu et al.[22] constructed a highly saturated linkage map for grape containing 70061 SNP markers in the integrated map. The construction of these linkage maps will prove beneficial for fine QTL mapping of important agronomic traits of these fruit trees in the future. Up to present, only two previous reports focused on genetic maps for hawthorn, one based on SRAP markers and the other based on 2b-RAD sequencing. The linkage maps constructed in these two reports were both for hawthorn cultivars ‘Damianqiu’ and ‘Qiujinxing’[23–24].

Compared with other woody fruit-trees, studies on high-density genetic mapping of hawthorn are extremely limited in terms of both quantity and quality; furthermore, QTL mapping related to important agronomic traits, such as fruit TD, VD, SFW, pericarp brittleness (PB), pericarp puncture hardness (PPH), and average sarcocarp firmness (ASF) of hawthorn has not been carried out; overall, this has greatly limited molecular-assisted breeding in hawthorn.

In this study, hawthorn cultivars ‘Shandongdamianqiu’ and ‘Xinbinruanzi’ were used as parental material for intraspecific hybridization. Both parents and 130 hybrid offspring were used for SNP detection based on RAD-seq. The objective of our study was to construct a high density genetic linkage map for hawthorn, and based on this genetic linkage map, we tried to conduct QTL mapping for six important agronomic traits which related to yield, dimensions, and storage ability of hawthorn berry. Based on our result, the research will lay a solid foundation for molecular-assisted selection of hawthorn for breeding purposes.

## Materials and methods

### Plant material and phenotype determination

Parents and hybrid offspring are obtained from the Hebei Normal University of Science and Technology (Qinhuangdao, China). Based on field observations over many years, female parent ‘Shandongdamianqiu’ and male parent ‘Xinbinruanzi,’ showed significant differences in berry dimension and firmness, yield, storage ability, and resistance to environmental stress. Crossings were conducted in 2010 and the seeds produced were sown in 2011; in all, 374 hybrid strains were grown, among which 130 hybrid individuals were randomly selected and used for the construction of a high-density maps and fruit phenotype detection. 1) SFW representing an average of 20 representative fruits with normal growth and development from the periphery of the crowns of 130 mapping population plants at fruit ripening was measured by an electronic balance. 2) Fruit TD and VD were measured by a Vernier caliper, and results are presented as the average of 20 berries. 3) PB, PPH, and ASF were determined with a texture analyzer (SMS model TA.XT Express Texture Tester).

Twenty fruits were selected from each of the 130 individuals. The diameter of the probe was 2 mm, and fruit puncture was carried out at a speed of 0.5 mm/s until a depth of 2 mm was reached by the probe. After puncture, fruits were returned to their initial position at a speed of 10 mm/s. PB represents the deformation distance of epidermal rupture and is inverse to the deformation. Generally, the smaller the deformation distance, the more obvious the PB. Pericarp resistance to puncture represents the maximum force applied during probe penetration through the pericarp. Average sarcocarp hardness represents the average force applied during probe penetration into the sarcocarp. Spearman correlations between traits were analyzed using SPSS16.0 software.

### Library construction for sequencing

Young, healthy leaves were collected from the two parents and the 130 selected individuals. The leaves were frozen in liquid nitrogen and stored at -80°C. Genomic DNA was extracted by a modified CTAB method [25]. DNA concentration and quality were detected using a NanoDrop 2000 spectrophotometer (Thermo Fisher Scientific, Waltham, MA, USA). TaqI restriction endonuclease (New England Biolabs, Ipswich, MA, USA) was used to digest qualified DNA of each individual and then barcoded P1 adapters (Illumina, USA) were ligated to the TaqI restriction site for each individual. Thereafter, samples were pooled in proportional amounts for shearing to an average size of 500 bp with a Bioruptor (Diagenode, Liège, Belgium). Sequencing libraries were constructed randomly with a total of 24 samples per library. Fragments ranging from 300 to 500 bp were extracted by 2% agarose gel electrophoresis and then ligated with the P2 adapter (San Diego, CA, USA). The constructed library was amplified by PCR with Phusion high-fidelity DNA polymerase (New England Biolabs), and the running conditions were: 98°C for 2 min, followed by 13 cycles at 98°C for 30 s, 60°C for 30 s, and 72°C for 15 s, and a final extension at 72°C for 5 min. Finally, samples of each selected individual were sequenced on an Illumina HiSeq^TM^ platform with the Illumina PE150 strategy.

### Development of SNP markers and construction of a genetic-linkage map

Raw reads were assigned to individual samples according to their nucleotide barcode using the axe pacakage [26]. Reads of low quality, including reads with >10% nucleotides with a quality value <30 (0.1% sequencing error), were trimming or discarded by fastp software [27]. Burrows-Wheeler Aligner software [28] was used to align clean reads from each sample against the reference genome (https://phytozome.jgi.doe.gov/pz/portal.html#!info?alias=Org_Mdomestica) under default mapping parameters. Molecular markers were called using the GATK pipeline, which considers indel-realignment and mark-duplication, and calls variants across all samples simultaneously through the HaplotypeCaller program in GATK 3.8.0 [29], the minimum minor allele frequency (MAF) was 0.05, and maximum call rate was 0.90.

The population type was cross pollinators (CP), representing a cross between two heterozygous diploid parents. There are five segregation types of CP populations (lm×ll, nn×np, hk×hk, ef×eg, and ab×cd), and three segregation types were genotyped. The marker code ‘lm×ll’ represents markers with first parent heterozygous and second parent homologous, ‘nn×np’ represents markers with the first parent homologous and the second parent heterozygous, and ‘hk×hk’ represents markers with both parents heterozygous. Considering genotyping error, the SNP markers were filtered by the following criteria: the expected segregation ratio of ‘lm×ll’ and ‘nn×np’ was 1:1, ‘hk×hk’ was 1:2:1; Chi-squared was calculated, and the threshold P-value was set to 0.05; The sequencing depth of the two parents was higher than 10×, and that of the offspring was higher than 5×; the lower depth genotype was set as missing. Finally, any locus with more than five missing data was filtered. The qualified markers were then used to construct paternal and maternal linkage maps using the JoinMap 4 software [30]. An LOD score cut-off of 5.0 was used to determine linkage groups. Map distances (cM) were converted using recombination frequencies through the Kosambi mapping function. The integrated map was generated by integrating the parental maps based on the shared markers using MergeMap [31–32] with a map weight a 1.0. The visualized linkage maps were subsequently drawn using MapChart 2.2 [33].

### QTL mapping of fruit phenotypic data

QTL mapping was conducted by combining fruit phenotype data and high-density integrated linkage map using QTL mapping software MapQTL 5.0.[34] Interval mapping strategy was used for QTL mapping analysis; QTLs were scanned in each linkage group at an interval of 1cM, QTLs were detected with a LOD threshold ≥3.0.[35–36] The percentage of variation was explained corresponding to the peak LOD in this study; software MapChart2.2 was used to draw the QTL interval[33]. The QTL region contains the marker related to peak LOD and its flanking markers which LOD ≥3.0.

## Results

### Fruit phenotype identification

**Fig 1:**
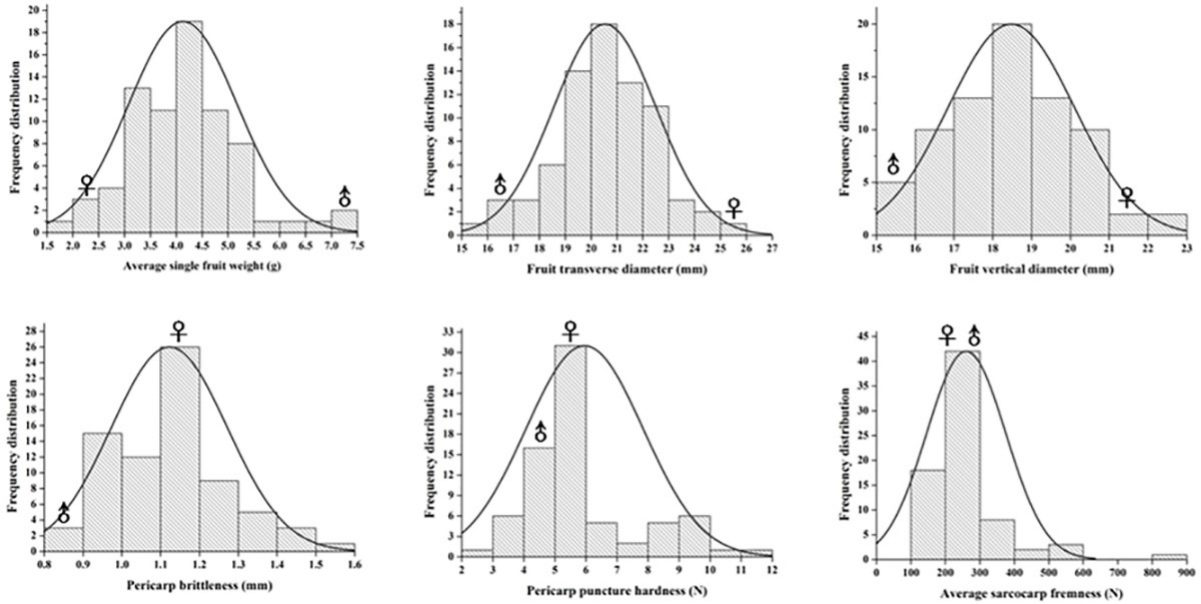
Phenotypic distribution of hawthorn hybrid population crossed from ‘Shandongdamianqiu’ and ‘Xinbinruanzi’. “Shandongdamianqiu” and “Xinbinruanzi’.

Fruit traits distribution is shown in Fig 1 and S1 Table. All phenotypic traits showed continuous variation and seemed to be typical QTLs. The average value of hybrid offspring fruit TD, VD and PB were 20.52mm, 18.47mm and 1.13mm, the minimum value were 15.27mm, 15.11mm and 0.86mm, the maximum value were 24.69mm, 22.75mm and 1.57mm; The value of SFW was ranged from 1.99g to 7.35g, and the average value was 4.11g; the value of fruit PPH and ASF were ranged from 2.47N to 11.84N and 0.03N to 8.18N, with the average value of 5.98N and 2.53N. Some transgressive segregation individuals were detected for each trait. Among them, PB, PPH, and average sarcocarp hardness were the most prominent. There was no significant correlation between average sarcocarp hardness, SFW and fruit VD. The correlation between fruit TD and SFW was the most significant (r = 0.960), followed by the correlation between fruit transverse and VD. In addition, there was a high correlation between PPH and average sarcocarp hardness (r = 0.792) and a significant negative correlation between PPH and PB (r = −0.651) (Table 1).

**Table 1:** Phenotypic correlation coefficients between the traits of hawthorn berries produced by crossing ‘Shandongdamianqiu’ with‘Xinbinruanzi’.

	TD	VD	SFW	PB	PPH	ASF
TD	1.00					
VD	0.832**	1.00				
SFW	0.960**	0.900**	1.00			
PB	-0.497**	-0.519**	-0.457**	1.00		
PPH	-0.432**	-0.338**	-0.296*	-0.651**	1.00	
ASF	-0.271*	-0.199	-0.169	0.330**	0.792**	1.00

Note: ‘TD’, ‘VD’, ‘SFW’, ‘PB’, ‘PPH’ and ‘ASF’ represent ‘fruit transverse diameter’, ‘fruit vertical diameter’, ‘single fruit weight’, ‘pericarp brittleness’, ‘pericarp puncture hardness’ and ‘average sarcocarp firmness’ respectively.

‘**’and ‘*’ represent significance at P<0.01 and P<0.05.

### Raw data analysis and development of SNP markers

**Fig 2:**
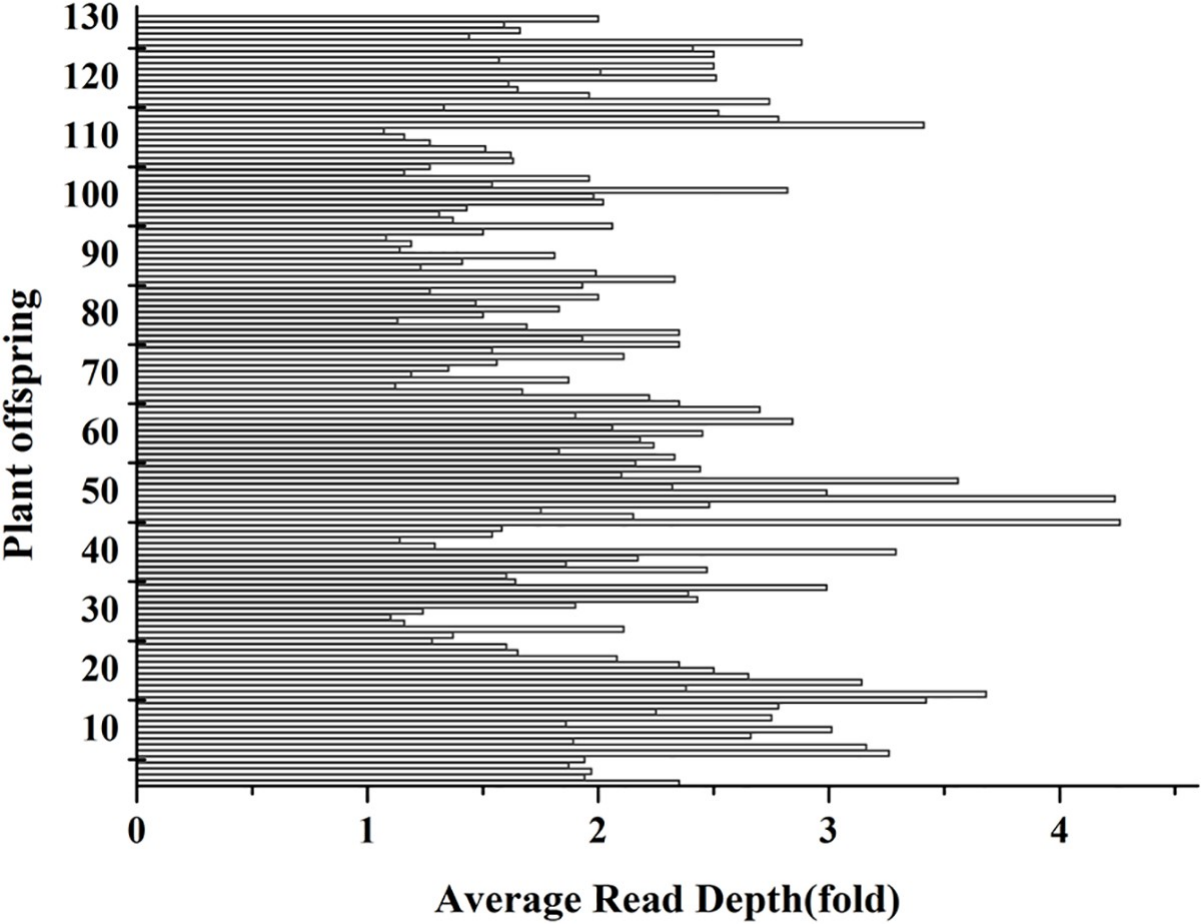
Clean reads number distribution of 130 hybrid offspring.

In all, 209.05 G data were obtained by analyzing the two parents and 130 hybrid offspring. Average data size was 1.58 G. In total, 708,798,612 clean reads were obtained, 13,116,028 for the female parent ‘Shandongdamianqiu’ and 13,305,74 for the male parent ‘Xinbinruanzi.’ The clean read distribution of the 130 hybrid offspring is shown in Fig 2.

**Fig 3:**
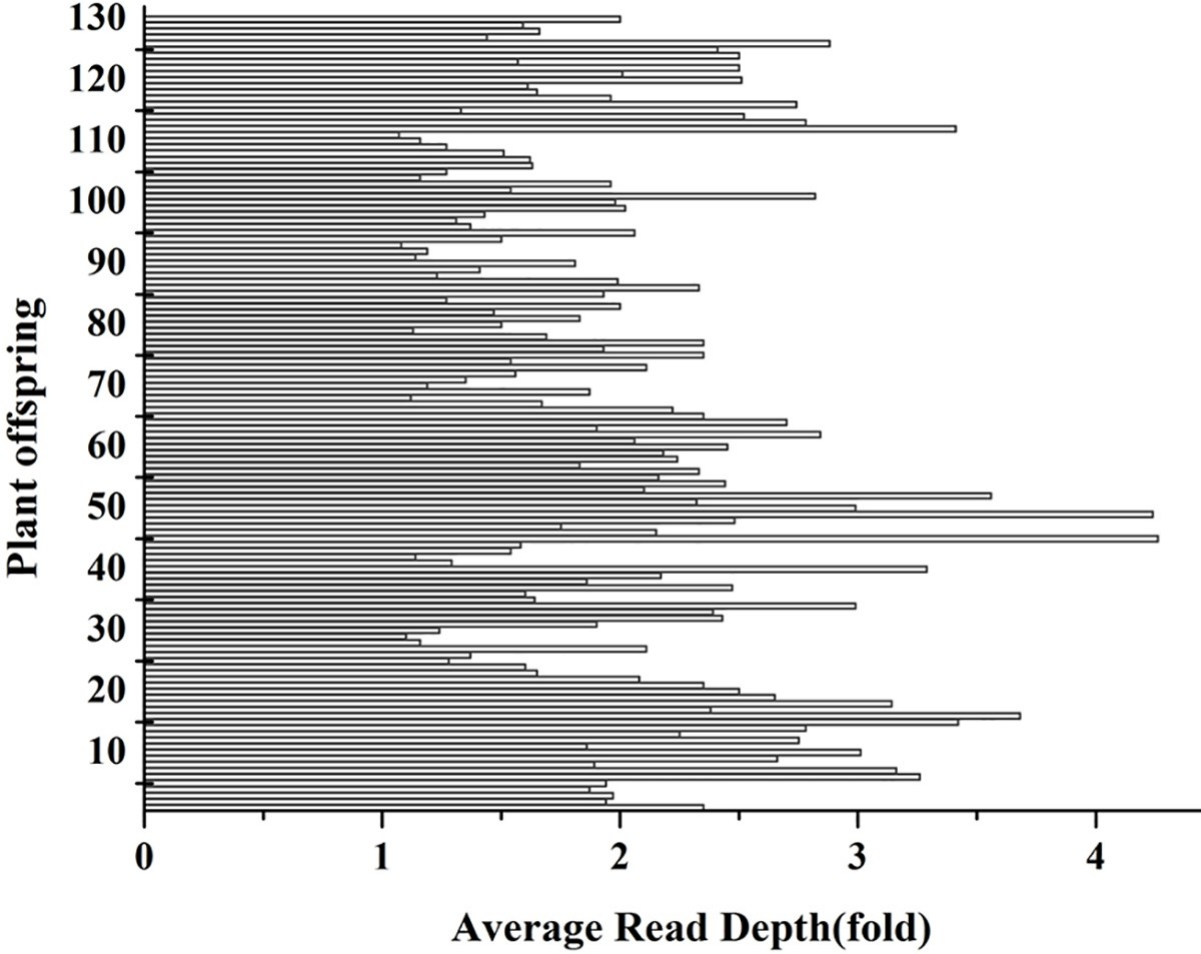
Sequencing depth distribution of 130 hybrid offspring.

**Fig 4:**
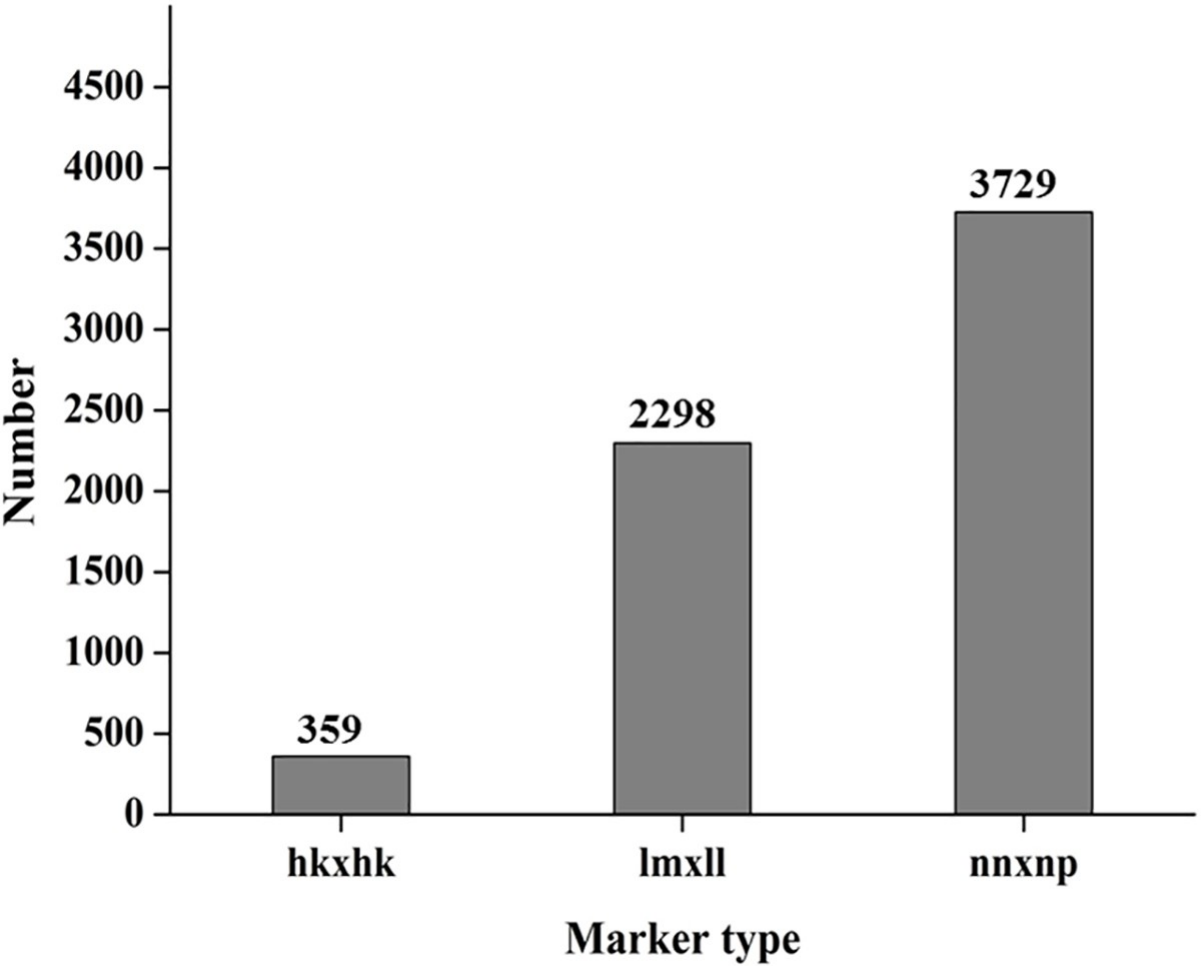
Number of different genotype markers. lm×ll represent the markers used for female map construction and the order was male×female, nn×np represent the markers used for male map construction and the order was female×male, hk×hk represent the markers contained by both of the parents.

The GC content and Q30 were 37.15% and 94.68%, respectively, for the female parent (‘Shandongdamianqiu’). For the male parent (‘Xinbinruanzi’), GC content and Q30 were 37.14% and 94.63%, respectively. The sequencing depths of ‘Shandongdamianqiu’ and ‘Xinbinruanzi’ were 5.07 and 5.14, respectively, and that of the 130 hybrid offspring is shown in Fig 3. After standard filtering, 6386 SNP markers were achieved and used to construct genetic linkage maps. The number of different genotype markers is shown in Fig 4 and S2 Table.

### Genetic-linkage map construction

Before constructing the integrated map, the maps for female and male parents were constructed, and the number of markers, distance between adjacent markers, and gaps in the same linkage group were determined. The female parent contained the marker with the type of lm×ll and hk×hk, male parent was constructed with the marker type nn×np and hk×hk (S3 Table). These two separate parental linkage maps were integrated into an integrated linkage map based on the common markers shared by the two parents (S4 Table).

For female parent ‘Shandongdamianqiu,’ 2657 SNP markers (containing 711 co-segregating markers and 276 marker bins) were distributed in 17 linkage groups with a total map length of 2689.65 cM. The length of the linkage group was between 109.34 and 217.77 cM. The shortest and longest linkage groups were LG8 and LG5, respectively. The number of markers per linkage group was between 22 and 237 (Table 2, S1 Fig). The average genetic distance of adjacent markers in these genetic maps was 1.01 cM. Among the 17 linkage groups, the average genetic distance of adjacent markers ranged from 0.60 to 5.91 cM. The percentage of Gap≤5cM in 17 linkage groups ranged between 55.00% and 97.87% (Table 3, S1 Fig).

**Table 2:** Marker distribution and total genetic length of 17 linkage groups.

Linkage group ID	Maker Number			Genetic distance(cM)		
	Female Map	Male map	Integrated map	Female Map	Male map	Integrated map
LG1	92	318	385	146.91	187.28	123.68
LG2	168	254	401	117.39	129.49	124.83
LG3	115	179	284	111.65	150.36	120.83
LG4	193	220	401	194.11	149.61	147.93
LG5	101	242	318	217.77	163.04	195.07
LG6	139	221	335	213.12	122.94	131.79
LG7	22	193	213	124.01	154.90	154.28
LG8	111	226	322	109.34	194.95	127.48
LG9	161	213	338	184.80	173.59	184.99
LG10	167	229	384	211.85	127.25	169.99
LG11	162	262	385	119.38	181.87	127.52
LG12	234	233	447	182.49	148.77	175.71
LG13	237	312	517	159.92	163.20	128.86
LG14	174	174	329	164.69	128.27	192.06
LG15	163	270	416	145.30	125.81	124.83
LG16	198	275	443	119.13	131.79	106.76
LG17	220	267	466	167.79	125.29	133.41
Total	2657	4088	6384	2689.65	2558.41	2470.02

**Table 3:** Average genetic distance of adjacent markers in 17 linkage groups.

Linkage group ID	Average genetic distance(cM)			Percentage of Gap≤5cM(Max Gap)		
	Female Map	Male map	Integrated map	Female Map	Male map	Integrated map
LG1	1.61	0.59	0.32	93.59%(12.17)	98.33%(8.59)	99.66%(6.03)
LG2	0.70	0.51	0.31	92.09%(11.03)	98.55%(9.80)	96.70%(10.04
LG3	0.98	0.84	0.43	89.80%(10.58)	96.60%(10.03)	95.73%(10.25)
LG4	1.01	0.68	0.37	94.27%(21.09)	95.63%(9.04)	98.79%(15.87)
LG5	2.18	0.68	0.62	91.21%(16.37)	97.60%(10.17)	98.21%(13.38)
LG6	1.54	0.56	0.39	92.24%(18.62)	97.63%(9.76)	98.48%(9.65)
LG7	5.91	0.81	0.73	55.00%(16.90)	98.73%(5.72)	98.86%(5.69)
LG8	0.99	0.87	0.40	88.76%(11.09)	97.14%(26.71)	96.84%(20.00)
LG9	1.16	0.82	0.55	94.24%(23.54)	94.38%(10.54)	97.44%(22.41)
LG10	1.27	0.56	0.44	93.75%(19.51)	98.92%(7.48)	99.06%(13.50)
LG11	0.74	0.70	0.33	92.54%(10.55)	97.06%(10.59)	98.37%(21.57)
LG12	0.78	0.64	0.39	95.96%(9.99)	97.96%(10.03)	98.94%(6.61)
LG13	0.68	0.52	0.25	97.46%(10.98)	99.19%(6.38)	99.76%(5.18)
LG14	0.95	0.74	0.59	97.01%(17.08)	97.28%(9.33)	98.50%(14.34)
LG15	0.90	0.47	0.30	97.20%(18.26)	100.00%(4.86)	99.72%(5.16)
LG16	0.60	0.48	0.24	93.17%(10.18)	99.52%(5.68)	97.42%(10.07)
LG17	0.77	0.47	0.29	97.87%(13.22)	99.53%(9.30)	99.74%(10.06)

Note: Percentage of Gap≤5cM represents (the number of intervals with less than5cM) / (total number of intervals).

A total of 4088 SNP markers (containing 1305 co-segregating markers and 483 marker bins) were anchored into 17 linkage groups of the male parent ‘Xinbinruanzi’ and the total genetic length was 2558.41 cM. The genetic length of each linkage group ranged from 122.94 to 194.95 cM; the longest was LG8 and shortest was LG6. Marker number of markers per linkage group ranged from 174 to 318; LG14 contained the smallest number and LG1 contained the largest number (Table 2, S2 Fig). Average genetic distance between adjacent markers in the 17 linkage groups was 0.63 cM; the longest average genetic distance between adjacent markers was LG8 and shortest were LG15 and LG17with average genetic distance of 0.87 and 0.47 cM, respectively. The percentage of Gap≤5cM in the 17 linkage groups ranged between 94.30% and 100.00% (Table 3, S2 Fig).

**Fig 5:**
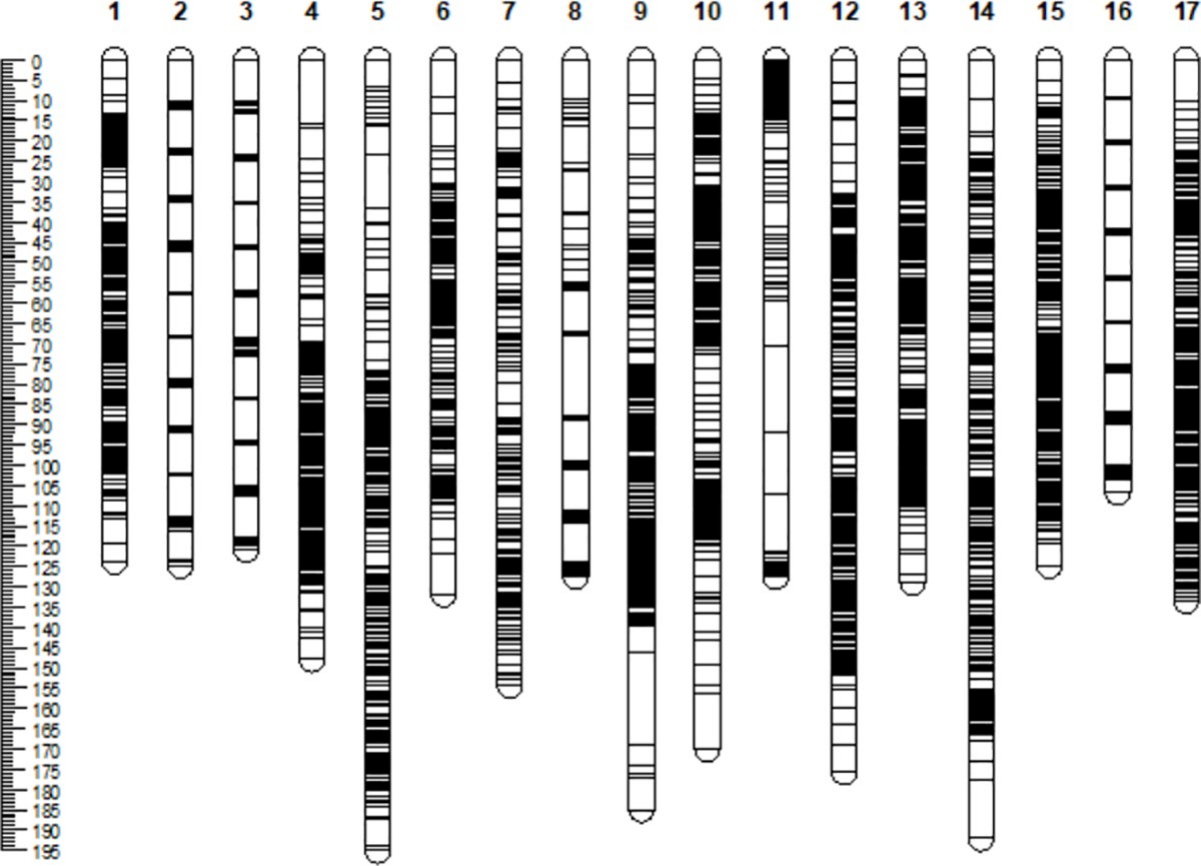
Marker distribution and genetic length of integrated map. Centimorgans (cM) indicated the genetic length of vertical scale. Black lines represent mapped markers. LG1–17 represents corresponding linkage groups.

All recombination events were integrated based on the 359 common molecular markers shared by the female and the male parents to obtain the integrated map. During the integration, 361 markers from female and male parent maps were dropped. Finally, a total of 6384 SNP markers(containing 1919 co-segregating markers and 732 marker bins) were anchored into 17 linkage groups of integrated map with a total map distance of 2470.02 cM. The genetic distance of each linkage group ranged between 106.76 and 195.07 cM, among which, the shortest and longest linkage groups were LG16 and LG5, respectively. The number of markers per linkage group ranged from 213 to 517; LG7 contained the smallest number and LG13 contained the largest number (Table 2, Fig 5). The average genetic distance between adjacent markers in the 17 linkage groups was 0.41 cM, the longest average genetic distance between adjacent markers was recorded for group LG7 and the shortest for group LG16 with average genetic distance of 0.73 and 0.24 cM, respectively. The percentage of Gap≤5cM in 17 linkage groups was between 95.73% and 99.76% (Table 3, Fig 5).

### QTL mapping

**Fig 6:**
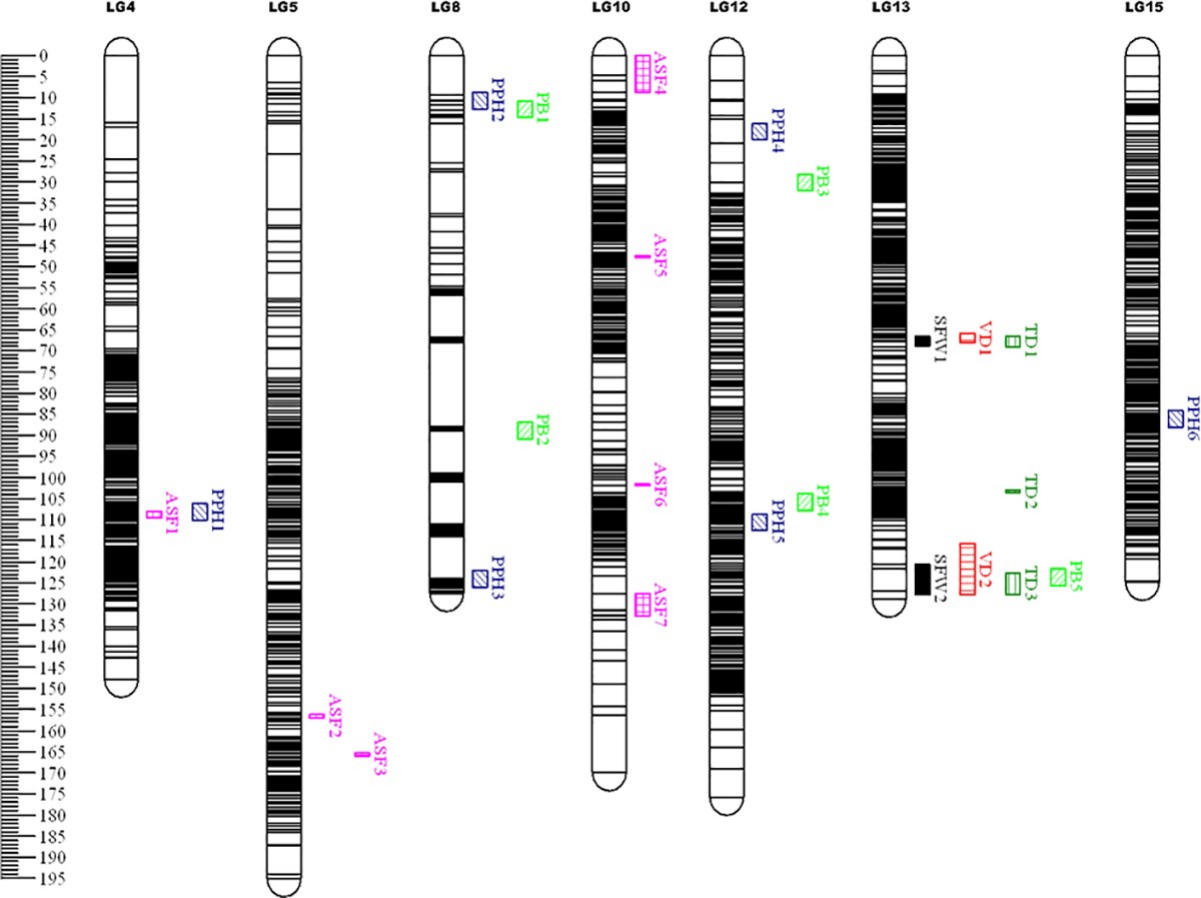
QTL mapping of hawthorn fruit traits. Different colors represent different traits. TD’, ‘VD’, ‘SFW’, ‘PB’, ‘PPH’ and ‘ASF’ represent ‘fruit transverse diameter’, ‘fruit vertical diameter’, ‘single fruit weight’, ‘pericarp brittleness’, ‘pericarp puncture hardness’ and ‘average sarcocarp firmness’ respectively.

A total of 25 QTLs were identified based on the integrated map and phenotypic data (Table 4 and Fig 6). Two QTLs were related to SFW in LG13 and were designated as SFW1 and SFW2. These explained 19.0–21.8% of the variance; the co-segregated molecular markers were chr5_12621976 and chr14_28230882; two more QTLs were related to fruit VD in LG13 and were designated as VD1 and VD2. These explained 18.1–24.3% of the variance and the co-segregated molecular markers were chr5_20336798 and chr14_28230882. Three QTLs were related to fruit TD in LG13 and were designated as TD1, TD2, and TD3; these explained 20.9%, 21.4%, and 19.4% of the variance, respectively. The co-segregated molecular markers were chr5_12621976, chr5_13033674, and chr12_30774038. Six QTLs were related to PPH; they were located in LG4, LG8, LG12, and LG15 and were named as PPH1, PPH2, PPH3, PPH4, PPH5, and PPH6. The variance they explained ranged from 17.7% to 25.2%. The co-segregated molecular markers were chr9_8217939, chr16_17501604, chr14_9820648, chr2_32713790, chr2_13241735, and chr11_33148988. Five more QTLs were related to PB and they were located in LG8, LG12, and LG13, and were named as PB1, PB2, PB3, PB4, and PB5. The variance they explained ranged from 18.1% to 32.2%. The co-segregated molecular markers were chr15_44435105, chr16_8716833, chr13_22602325, chr2_11966150, and chr14_28230882. Additionally, seven QTLs were related to ASF and they were located in LG4, LG5, and LG10 and named as ASF1, ASF2, ASF3, ASF4, ASF5, ASF6, and ASF7, and the variance they explained ranged from 19.3% to 35.0%. The co-segregated molecular markers were chr9_4803043, chr13_1302262, chr13_467216, chr12_25140899, chr12_26705309, chr12_23919622, and chr12_25334013.

**Table 4:** QTL mapping results for hawthorn fruit trait of 'Shandongdamianqiu'×'Xinbinruanzi'.

Traits	QTL location	LG	Position (cM)	Co-segregated marker	Peak LOD	*R*^*2*^(%)	allele configuration^a^
SFW	SFW1	LG13	67.58	chr5_12621976	3.34	19.0	lm×ll
	SFW2	LG13	124.66	chr14_28230882	3.9	21.8	nn×np
VD	VD1	LG13	66.17	chr5_20336798	3.17	18.1	hk×hk
	VD2	LG13	123.66	chr14_28230882	4.42	24.3	nn×np
TD	TD1	LG13	67.58	chr5_12621976	3.71	20.9	lm×ll
	TD2	LG13	90.73	chr5_13033674	3.83	21.4	lm×ll
	TD3	LG13	125.66	chr12_30774038	3.42	19.4	nn×np
PPH	PPH1	LG4	108.12	chr9_8217939	3.11	18.0	nn×np
	PPH2	LG8	10.84	chr16_17501604	3.85	21.8	hk×hk
	PPH3	LG8	123.98	chr14_9820648	4.45	25.2	nn×np
	PPH4	LG12	18.03	chr2_32713790	3.89	22.0	lm×ll
	PPH5	LG12	110.60	chr2_13241735	4.12	23.2	lm×ll
	PPH6	LG15	86.23	chr11_33148988	3.04	17.7	nn×np
PB	PB1	LG8	12.81	chr15_44435105	3.13	18.1	lm×ll
	PB2	LG8	88.86	chr16_8716833	4.97	27.2	nn×np
	PB3	LG12	30.11	chr13_22602325	5.56	29.9	lm×ll
	PB4	LG12	105.80	chr2_11966150	6.07	32.2	nn×np
	PB5	LG13	123.66	chr14_28230882	3.42	19.7	nn×np
ASF	ASF1	LG4	108.27	chr9_4803043	3.45	19.8	lm×ll
	ASF2	LG5	156.86	chr13_1302262	3.5	20.1	nn×np
	ASF3	LG5	165.64	chr13_467216	6.73	35.0	nn×np
	ASF4	LG10	8.00	chr12_25140899	3.48	20.0	lm×ll
	ASF5	LG10	47.54	chr12_26705309	3.35	19.3	nn×np
	ASF6	LG10	101.51	chr12_23919622	3.43	19.7	lm×ll
	ASF7	LG10	131.71	chr12_25334013	3.63	20.7	lm×ll

Note: ‘TD’, ‘VD’, ‘SFW’, ‘PB’, ‘PPH’ and ‘ASF’ represent ‘fruit transverse diameter’, ‘fruit vertical diameter’, ‘single fruit weight’, ‘pericarp brittleness’, ‘pericarp puncture hardness’ and ‘average sarcocarp firmness’ respectively. ^a^The allele configuration (male× female) of nearest marker as coded in JoinMap.

## Discussion

### Selection of parents and raising of the hybrid population for RAD-seq

As a key step in genetic-linkage map construction, the female and male parent to use for the construction of the mapping population is very important and the fertility and genotype separation within the hybrid offspring should also be taken into account. Therefore, parents with significant genetic differences should be preferred. In this study, these two factors were both taken into account. For female parent ‘Shandongdamianqiu,’ berries were oblate, larger, and the average fruit weight was about 15 g; further, fruit texture and seeds were very hard and showed good storability. Additionally, ‘Shandongdamianqiu’ showed higher environmental adaptability and resistance to a variety of pests and diseases. In contrast, fruit size of male parent ‘Xinbinruanzi’ was smaller, with average fruit weight about 1.5 g, fruit texture and seeds were very soft and thereby, vulnerable to many pests and diseases. A large number of F1 generations were produced by the hybridization of the two parents, indicating a good interspecific hybridization affinity. Further, many fruit traits showed significant differences. Therefore, the specific, constructed hybrid population may provide an important source of material for genetic and QTL mapping research of quantitative traits of hawthorn.

### The advantage of SNP marker and high density linkage map construction for hawthorn

A variety of molecular markers can be used to construct a genetic map in botanical studies, such as RAPD, RFLP, SRAP, AFLP, and SSR [37–48]. However, the efficiency of linkage-map construction by the traditional molecular markers is not very high [49] Furthermore, the limited number of markers in these genetic maps may affect their application. Compared with the molecular markers mentioned above, SNP markers are more widely and uniformly distributed in most species, with high genetic stability and accuracy. Moreover, with the development of NGS technology, the difficulties encountered in large-scale SNP development have been solved [50–51]. High-density linkage map construction in our study indicated that RAD-seq can be applied to develop large-scale SNP markers and construct high-density molecular genetic maps for some species without reference genome, and in the same time greatly shortens the sequencing cycle and reduces the cost of marker development.

The construction of a high-density genetic map is the key step for QTL mapping of quantitative traits and has been widely used in studies on many plants and animals [16,52–55]. To date, there are only two reports on genetic map construction in hawthorn. Wang et al. [23] first constructed the genetic maps for hawthorn cultivars ‘Damianqiu’ and ‘Qiujinxing’ by using SRAP markers and 92 hybrid offspring. However, the number of markers in these genetic maps was very low, and the average genetic distance between adjacent markers was very large, which was not suitable for fine QTL mapping. Furthermore, SRAP markers also showed poor repeatability and stability in linkage map construction [23] and not considerable for constructing genetic maps of other hawthorn cultivars. Zhao et al. constructed other genetic maps by using 107 hybrid offspring of the same population and 2b-RAD technology and a total of 3894 SNP markers were achieved in the integrated map [24]. Compared with the genetic maps constructed by SRAP markers, marker number and density were significantly improved, while the 2b-RAD tags used for SNP marker detection were only 33 bp, which was not conducive to primer design of selected markers.

In this study, the RAD tags used for SNP detection were 150 bp, and the number of hybrid offspring was 130. The genetic distance of the female parent map was 2689.65 cM, containing 2656 SNP markers; conversely, the genetic distance of the male parent map was 2558.41 cM, containing 4085 SNP markers. Compared with the previously reported maps, the number of markers and the map quality were greatly improved.

Although there remain many larger gaps in some linkage groups, this may be caused by an insufficient number of polymorphisms of markers, missing data, or heterozygosity of the parents [56–59]. Therefore, measures should be taken in the future to further narrow these regions, such as increasing the number of sequenced individuals and sequencing depth.

### QTL mapping and QTLs co-location

In this study, we found that two or more QTLs related to different traits were located in the same region, such as QTLs PPH1 and ASF1, which were related to PPH and ASF and were both located in the same region in LG4. QTLs PPH2 and PB1, which were related to PPH and PB, were co-located in LG8. TD1, VD1, and SFW1, which were related to fruit TD, VD, and SFW, were co-located in LG13. Similarly, PB5, TD3, VD2, and SFW2 were also co-located in LG13 (Fig 6). In our study, only three QTLs showed high LOD values (>5.0), while the majority were near the threshold. This may be due to drawbacks [60–61] of IM strategy when used for QTL mapping. However, some QTLs related to fruit TD, VD, and average SFW were closely mapped and consistent in our study. These results can somewhat validate the reliability of our QTL results.

For berry traits QTL research, Wu et al., 2014 conducted QTL mapping for pear fruit TD, VD and SFW, interestingly, they found that QTLs related to these three traits were co-located in LG17 and the overlapping region was ranged from 11.3cM-25.6cM [20], this was similar with our result associated with hawthorn fruit TD, VD and SFW. Until now, there is no report about QTL mapping of fruit pericarp brittleness, pericarp puncture hardness and average sarcocarp firmness. For the co-located QTLs, similar results were reported in a study of glucose and fructose content in grapes by Chen et al.,[52] who found that the QTLs which related these two traits were co-located in LG14 of the grape linkage map. The correlation between glucose and fructose content was very high. In our study, some of the traits were also highly correlated (Table 1). For example, fruit TD, VD, and SFW were more highly positively correlated, and some QTLs that were relative to these three traits were co-located in LG13. PPH and ASF were also highly positively correlated, and some QTLs related to these two traits were also co-located in LG4. Besides, PB and PPH were highly negatively correlated, and some QTLs related to these two traits were co-located in LG8. PB was significantly negatively correlated with fruit TD, VD, and SFW, and the QTL PB5 was co-located with TD3, VD2, and SFW2. This may because these traits are regulated by the same unique gene. For example, gene GW2, which encodes E3 ubiquitin ligase in rice, can simultaneously regulate grain size, yield, and spike length [62]; similarly, gene DWARF27 encoding an iron-containing protein, can simultaneously regulate tiller number and plant height in rice [63]. In *Arabidopsis thaliana*, flowering-related gene CO can simultaneously regulate the time of lignin extension [64]. QTLs related to fruit VD, SFW, and ASF were not found to be co-located, and the correlations between them were not significant.

In total of 61 annotated genes were selected according to our QTL mapping result, the annotated gene number related to the six traits was ranged from 2 to 22 (S5 Table). Among these genes, pectinesterase gene was preliminarily selected as the candidate gene. Previous researches have reported its function in fruit development and firmness [65–66]. The transcription factor family *MYB*, *ERF*, *NAC* and *WRKY* were widely exist in plant, and played important role in plant growth and development [67–71]. In our study, some transcription factors were selected based on our QTL result, including *MYB114*, *ABR1*, *NAC71* and *WRKY33*. Until now, these is no research reported their role in fruit development and firmness, our result may provide a reference for functional study of these genes in the future.

## Conclusions

Compared with the hawthorn genetic maps previously reported, the number of SNP markers was greatly improved in this study, and to our knowledge, this is the highest density genetic map of hawthorn ever constructed. This achievement lays a solid foundation for fine QTL mapping of important agronomic traits of hawthorn in the future.

In this study, we first performed QTL mapping for hawthorn fruit traits including, SFW, fruit transverse and VDs, PB, PPH and average sarcocarp hardness. A number of QTLs related to these traits were found. To ensure the accuracy of the QTLs achieved, we will continue to assess these traits for years to come and then conduct QTL mapping based on the constructed genetic map. There are no studies on QTL mapping for important agronomic traits of hawthorn. Therefore, this study will be an important reference for molecular-assisted selection of hawthorn for breeding in the future.

## Supporting information

S1 Fig Marker distribution and genetic length in 17 linkage groups of female parent ‘Shandongdamianqiu’. (PDF)

S2 Fig Marker distribution and genetic length in 17 linkage groups of male parent ‘Xinbinruanzi’. (PDF)

S1 Table Hawthorn phenotypic trait identificated in this study. (XLSX)

S2 Table Genotype information of the SNP markers. (XLSX)

S3 Table Marker names types and map positions statement for female, male and integrated map. (XLSX)

S4 Table Common markers shared by the two parents. (XLSX)

S5 Table Gene statistics based on QTL mapping result. (XLSX)

## References

[1] Verma SK, Jain V, Verma D, Khamesra A. *Crataegus oxyacantha*–a cardioprotective herb. J. Herb. Med. Toxicol. 2007; 1: 65–71.

[2] Yang B, Liu P. Composition and health effects of phenolic compounds in hawthorn (*Crataegus spp*.) of different origins. J. Sci. Food. Agric. 2012; 92:1578–1590. 10.1002/jsfa.5671 22488722

[3] Liu PZ, Yang BR, Kallio H. Characterization of phenolic compounds in Chinese hawthorn (*Crataegus pinnatifida Bge.* var.major) fruit by high performance liquid chromatography electrospray ionization mass spectrometry. Food Chem. 2010; 121:1188–1197. 10.1016/j.foodchem.2010.02.002 25214140

[4] Cao GY, Feng YX, Qin XQ. Analysis of the chemical constituents of hawthorn fruits and their quality evaluation. Acta Pharmacol. Sin. 1995; 30:138–143. 10.16438/j.0513-4870.1995.02.012

[5] Özcan M, Hacıseferoğulları H, Marakoğlub T, Derya Arslana D. Hawthorn (*Crataegus* spp.) fruit: some physical and chemical properties. J. Food Eng. 2005; 69: 409–413. 10.1016/j.jfoodeng.2004.08.032

[6] Wu J, Peng W, Qin R, Zhou H. *Crataegus pinnatifida*: chemical constituents, pharmacology, and potential applications. Molecules. 2014; 19:1685–1712. 10.3390/molecules19021685 24487567 PMC6271784

[7] Liu P, Kallio H, Lü D, Zhou C, Yang B. Quantitative analysis of phenolic compounds in Chinese hawthorn (*Crataegus* spp.) fruits by high performance liquid hromatography-electrospray ionisation mass spectrometry. Food Chem. 2011; 127: 1370–1377. 10.1016/j.foodchem.2011.01.103 25214140

[8] Chai WM, Chen CM, Gao YS, Feng HL, Ding YM, Shi Y, et al Structural analysis of proanthocyanidins isolated from fruit stone of Chinese hawthorn with potent antityrosinase and antioxidant activity. *J*. *Agric*. *Food Chem*. 62, 123–129 (2014). 10.1021/jf405385j 24313351

[9] Tanksley SD, Bernachi D, Beck-Bunn T, Emmatty D, Eshed Y, Inai S, et al Yield and quality evaluations on a pair of processing tomato lines nearly isogenic for the Tm2a gene for resistance to the tobacco mosaic virus. Euphytica.1998; 99: 77–83. 10.1023/a:1018320232663

[10] Hohenlohe PA, Amish SJ, Catchen JM, Allendorf FW, Luikart G. Next-generation RAD sequencing identifies thousands of SNPs for assessing hybridization between rainbow and westslope cutthroat trout. Mol. Ecol. Resour. 2011; 1:117–122. 10.1111/j.1755-0998.2010.02967.x 21429168

[11] Hua Y, Wei CL, Liu HW, Wu JL, Li ZG, Zhang L, et al Genetic Divergence between *Camellia sinensis* and Its Wild Relatives Revealed via Genome-Wide SNPs from RAD Sequencing. Plos One. 2016; 11: e0151424 10.1371/journal.pone.0151424 26962860 PMC4786323

[12] Brookes AJ. The essence of SNPs. Gene. 1999 234:177–186. 10.1016/s0378-1119(99)00219-x 10395891

[13] Fujii H, Shimada T, Nonaka K, Kita M, Kuniga T, Endo T, et al High-throughput genotyping in citrus accessions using an SNP genotyping array. Tree Genet. Genomes. 9, 145–153 (2013). 10.1007/s11295-012-0542-3

[14] Emerson KJ, Merz CR, Catchen JM, Hohenlohe PA, Cresko WA, Bradshaw WE, et al Resolving postglacial phylogeography using high-throughput sequencing. PNAS. 2010; 107:16196–16200. 10.1073/pnas.1006538107 20798348 PMC2941283

[15] Pfender WF, Saha MC, Johnson EA, Slabaugh MB. Mapping with RAD (restriction-site associated DNA) markers to rapidly identify QTL for stem rust resistance in *Lolium perenne*. Theor. Appl.Genet. 2011; 122:1467–1480. 10.1007/s00122-011-1546-3 21344184

[16] Baird NA, Etter PD, Atwood TS, Currey MC, Shiver AL, Lewis ZA, et al Rapid SNP discovery and genetic mapping using sequenced RAD markers. PLoS One. 2008; 3: e3376 10.1371/journal.pone.0003376 18852878 PMC2557064

[17] Baxter SW, Davey JW, Johnston JS, Shelton AM, Heckel DG, Jiggins CD, et al Linkage mapping and comparative genomics using next-generation RAD sequencing of a non-model organism. PLoS One. 2011; 6:e19315 10.1371/journal.pone.0019315 21541297 PMC3082572

[18] Wang L, Xia Q, Zhang Y, Zhu X, Zhu X, Li D, et al Updated sesame genome assembly and fine mapping of plant height and seed coat color QTLs using a new high-density genetic map. BMC Genomics. 2016; 17:31 10.1186/s12864-015-2316-4 26732604 PMC4702397

[19] Sun R, Chang Y, Yang F, Wang Y, Li H, Zhao Y, et al A dense SNP genetic map constructed using restriction site-associated DNA sequencing enables detection of QTLs controlling apple fruit quality. BMC Genomics. 2015; 16:747 10.1186/s12864-015-1946-x 26437648 PMC4595315

[20] Wu J, Li LT, Li M, Awais K, Li XG, Chen H, et al High-density genetic linkage map construction and identification of fruit-related QTLs in pear using SNP and SSR markers. *J. Exp. Bot*. 2014; 65: 5771–5781. 10.1093/jxb/eru311 25129128 PMC4203118

[21] Zhao J, Jian J, Liu G, Wang J, Lin M, Ming Y, et al Rapid SNP Discovery and a RAD-Based High-Density Linkage Map in Jujube (*Ziziphus Mill*.). PLoS One. 2014; 9: e109850 10.1371/journal.pone.0109850 25303289 PMC4193841

[22] Zhu JC, Guo YS, Su K, Liu ZD, Ren ZH, Li K, et al Construction of a highly saturated Genetic Map for *Vitis* by Next-generation Restriction Site-associated DNA Sequencing. BMC Plant Biol. 2018; 18:347 10.1186/s12870-018-1575-z 30541441 PMC6291968

[23] Wang G, Guo Y, Zhao YH, Su K, Zhang JJ. Construction of a molecular genetic map for hawthorn based on SRAP markers. Biotechnol. Biotec. Eq. 2015; 29:1–7. 10.1080/13102818.2015.1018322

[24] Zhao YH, Su K, Wang G, Zhang L, Zhang J, Li J, et al High-Density Genetic Linkage Map Construction and Quantitative Trait Locus Mapping for Hawthorn (*Crataegus pinnatifida* Bunge). Sci. Rep. 2017; 7:5492 10.1038/s41598-017-05756-5 28710433 PMC5511184

[25] Hanania U, Velcheva M, Sahar N, Perl A. An improved method for isolating high-quality DNA from *Vitis vinifera* nuclei. Plant Mol. Biol. Rptr. 2004; 22, 2173–177. 10.1007/bf02772724

[26] Murray KD, Borevitz J O. Axe: rapid, competitive sequence read demultiplexing using a trie. Bioinformatics. 2018; 34: 3924–3925. 10.1093/bioinformatics/bty432 29868827

[27] Chen S, Zhou Y, Chen Y, Gu J. fastp: an ultra-fast all-in-one FASTQ preprocessor. Bioinformatics. 2018; 34, 884–890. 10.1093/bioinformatics/btx692 30423086 PMC6129281

[28] Li H, Durbin R. Fast and accurate short read alignment with burrows-wheeler transform. Bioinformatics. 2009; 25:1754–1760. 10.1093/bioinformatics/btp324 19451168 PMC2705234

[29] McKenna A, Hanna M, Banks E, Sivachenko A, Cibulskis K, Kernytsky A, et al The genome analysis toolkit: a map reduce framework for analyzing next-generation DNA sequencing data. Genome Res. 2014; 20:1297–1303. 10.1101/gr.107524.110.20 PMC2928508 20644199

[30] Van Ooijen JW. JoinMap 4. Software for the calculation of genetic linkage maps in experimental populations. Kyazma BV, Wageningen, Netherlands; 2006.

[31] Kakioka R, Kokita T, Kumada H, Watanabe K, Okuda N A. RAD-based linkage map and comparative genomics in the gudgeons (genus *Gnathopogon*, Cyprinidae). Bmc Genomics. 2013; 14:1–11. 10.1186/1471-2164-14-1 23324215 PMC3583795

[32] Wu Y, Close TJ, Lonardi S. On the accurate construction of consensus genetic maps. Comput Syst Bioinformatics Conf. 2008; 7: 285–296 (2008). 10.1142/9781848162648_0025 19642288

[33] Voorrips RE. MapChart: software for the graphical presentation of linkage maps and QTLs. J. Hered. 2002; 93:77–78 (2002). 10.1093/jhered/93.1.77 12011185

[34] van Ooijen JW. MapQTL^®^ 5. Software for the Mapping of Quantitative Trait Loci in Experimental Populations. Kyazma BV, Wageningen 2004.

[35] Fischer BM, Salakhutdinov I, Akkurt M, Eibach R, Edwards KJ, Töpfer R, et al Quantitative trait locus analysis of fungal disease resistance factors on a molecular map of grapevine. Theor. Appl. Genet. 2004; 108:501–515. 10.1007/s00122-003-1445-3 14574452

[36] Lin H. Leng H, Guo YS, Kondo S, Zhao YH, Shi GL, et al. QTLs and candidate genes for downy mildew resistance conferred by interspecific grape (*V. vinifera* L. × *V. amurensis* Rupr.) crossing. Sci. Hortic. 2019; 244: 200–207. 10.1016/j.scienta.2018.09.045.

[37] Dalbó MA, Ye GN, Weeden NF, Steinkellner H, Sefc KM, Reisch BI. A gene controlling sex in grapevines placed on a molecular marker-based genetic map. Genome. 2000; 43:333–338. 10791822

[38] Doligez A, Bouquet A, Danglot Y, Lahogue F, Riaz S, Meredith P. Genetic mapping of grapevine (*Vitis vinifera* L.) applied to the detection of QTLs for seedlessness and berry weight. Theor. Appl.Genet. 2002; 105: 80–795. 10.1007/s00122-002-0951-z 12582493

[39] Grando MS, Bellin D, Edwards KJ, Pozzi C, Stefanini M, Velasco R. Molecular linkage maps of *Vitis vinifera* L. and *Vitis riparia* Mchx. Theor. Appl.Genet. 2003;106:12–13. 10.1007/s00122-002-1170-3 12748772

[40] Doucleff M, Jin Y, Gao F, Riaz S, Krivanek AF, Walker MA. A genetic linkage map of grape, utilizing *Vitis rupestris* and *Vitis arizonica*. Theor. Appl.Genet. 2004; 109:1178–1187. 10.1007/s00122-004-1728-3 15292989

[41] Riaz S, Dangl GS, Edwards KJ, Meredith CP. A microsatellite marker based framework linkage map of *Vitis vinifera* L. Theor. Appl.Genet. 2004; 108: 864–872. 10.1007/s00122-003-1488-5 14605808

[42] Riaz S, Krivanek AF, Xu K, Walker MA. Refined mapping of the Pierce's disease resistance locus, *PdR1*, and Sex on an extended genetic map of *Vitis rupestris* x *V*. *arizonica*. Theor. Appl.Genet. 2006, 113:1317–1329. 10.1007/s00122-006-0385-0 16960717

[43] Adam-Blondon A F, Roux C, Claux D, Butterlin G, Merdinoglu D, This P. Mapping 245 SSR markers on the *Vitis vinifera* genome: a tool for grape genetics. Theor. Appl.Genet. 2004,109: 1017–1027. 10.1007/s00122-004-1704-y 15184982

[44] Lowe KM, Walker MA. Genetic linkage map of the interspecific grape rootstock cross Ramsey (*Vitis champinii*) x Riparia Gloire (*Vitis riparia*). Theor. Appl.Genet.2006; 112:1582–1592. 10.1007/s00122-006-0264-8 16607514

[45] Vezzulli S, Troggio M, Coppola G, Jermakow A, Cartwright D, Zharkikh A, et al A reference integrated map for cultivated grapevine (*Vitis vinifera* L.) from three crosses, based on 283 SSR and 501 SNP-based markers. Theor. Appl.Genet. 2008; 117:499–511. 10.1007/s00122-008-0794-3 18504538

[46] Salmaso M, Malacarne G, Troggio M, Faes G, Stefanini M, Grando MS, et al A grapevine (*Vitis vinifera* L.) genetic map integrating the position of 139 expressed genes. Theor. Appl.Genet. 2008;116:1129–1143. 10.1007/s00122-008-0741-3 18347774

[47] Marguerit E, Boury C, Manicki A, Donnart M, Butterlin G, Némorin A, et al Genetic dissection of sex determinism, inflorescence morphology and downy mildew resistance in grapevine. Theor. Appl.Genet. 2009; 118:1261–1278. 10.1007/s00122-009-0979-4 19238349

[48] Zhang J, Hausmann L, Eibach R, Welter LJ, Töpfer R, Zyprian EM. A framework map from grapevine V3125 (*Vitis vinifera* ‘Schiava grossa’×Riesling’)×rootstock cultivar ‘Börner’ (*Vitis riparia*×*Vitis cinerea*) to localize genetic determinants of phylloxera root resistance. Theor. Appl.Genet. 2009; 119:1039–1051. 10.1007/s00122-009-1107-1 19626311

[49] Huang XH, Feng Q, Qian Q, Zhao Q, Wang L, Wang A, et al High-throughput genotyping by whole-genome resequencing. Genome Res. 2009; 19:1068–1076. 10.1101/gr.089516.108 19420380 PMC2694477

[50] Divne AM, Allen M. A DNA microarray system for forensic SNP analysis. Forensic Sci Int. 2005; 154:111–121. 10.1016/j.forsciint.2004.09.134 16182957

[51] Vossen RH, Aten E, Roos A, den Dunnen JT. High-resolution melting analysis (HRMA): more than just sequence variant screening. Hum Mutat. 2009; 30:860–866. 10.1002/humu.21019 19418555

[52] Chen J, Wang N, Fang LC, Liang ZC, Li SH, Wu BH. Construction of a high-density genetic map and QTLs mapping for sugars and acids in grape berries. BMC Plant Biol. 2015; 15:28 10.1186/s12870-015-0428-2 25644551 PMC4329212

[53] Wang YK, Ning Z, Hu Y, Chen J, Zhao R, Chen H, et al Molecular Mapping of Restriction-Site Associated DNA Markers in Allotetraploid Upland Cotton. PLoS One. 2015; 10: e0124781 10.1371/journal.pone.0124781 25894395 PMC4403916

[54] Houston RD, Davey JW, Bishop SC, Lowe NR, Mota-Velasco JC, Hamilton A, et al Characterisation of QTL-linked and genome-wide restriction site-associated DNA (RAD) markers in farmed *Atlantic salmon*. BMC Genomics, 2012; 13: 244 10.1186/1471-2164-13-244 22702806 PMC3520118

[55] Sapkota S, Chen LL, Yang S, Hyma KE, Cadle-Davidson L, Hwang CF, et al Construction of a high-density linkage map and QTL detection of downy mildew resistance in *Vitis aestivalis*-derived ‘norton’. Theor. Appl.Genet. 2018; 132:137–147. 10.1007/s00122-018-3203-6 30341491

[56] Liu D, Ma C, Hong W, Huang L, Liu M, Liu H, et al Construction and analysis of high-density linkage map using high-throughput sequencing data. PLoS One. 2014; 9: e98855 10.1371/journal.pone.0098855 24905985 PMC4048240

[57] Shao C, Niu Y, Rastas P, Liu Y, Xie Z, Li H, et al Genome-wide SNP identification for the construction of a high-resolution genetic map of Japanese flounder (*Paralichthys olivaceus*): applications to QTL mapping of *Vibrio anguillarum* disease resistance and comparative genomic analysis. DNA Res. 2015; 22:161–170. 10.1093/dnares/dsv001 25762582 PMC4401326

[58] Sun X, Liu D, Zhang X, Li W, Liu H, Hong W, et al SLAF-seq: an efficient method of large-scale de novo SNP discovery and genotyping using high-throughput sequencing. PLoS One. 2013; 8:e58700 10.1371/journal.pone.0058700 23527008 PMC3602454

[59] Wu J, Li LT, Li M, Khan MA, Li XG, Chen H, et al High-density genetic linkage map construction and identification of fruit-related QTLs in pear using SNP and SSR markers. J. Exp. Bot. 2014; 65:5771–5781. 10.1093/jxb/eru311 25129128 PMC4203118

[60] Zeng ZB. Theoretical basis for separation of multiple linked gene effects in mapping quantitative trait loci. PNAS. 1993; 90:10972–10976. 10.1073/pnas.90.23.10972 8248199 PMC47903

[61] Zeng ZB. Precision mapping of quantitative trait loci. Genetics. 1994; 136: 1457–1468. 10.1007/s00122-012-2032-2 8013918 PMC1205924

[62] Song XJ, Huang W, Shi M, Zhu MZ, Lin HX. A QTL for rice grain width and weight encodes a previously unknown RING-type E3 ubiquitin ligase. Nat.Genet. 2007;39:623–630. 10.1038/ng2014 17417637

[63] Lin H, Wang R, Qian Q, Yan M, Meng X, Fu Z, et al DWARF27, an Iron-Containing Protein Required for the Biosynthesis of Strigolactones, Regulates Rice Tiller Bud Outgrowth. Plant Cell. 2009; 21:1512–1525. 10.1105/tpc.109.065987 19470589 PMC2700539

[64] Sibout R, Plantegenet S, Hardtke CS. Flowering as a Condition for Xylem Expansion in Arabidopsis Hypocotyl and Root. Curr. Biol. 2008; 18: 458–463. 10.1016/j.cub.2008.02.070 18356049

[65] Johnston JW, Hewett EW, Hertog M. Postharvest softening of apple (*Malus domestica*) fruit: a review. New Zealand journal of Crop Hortic Science. 2002; 30:145–160. 10.1080/01140671.2002.9514210

[66] Iwanami H, Moriya S, Kotoda N, Abe K. Turgor closely relates to postharvest fruit softening and can be a useful index to select a parent for producing cultivars with good storage potential in apple. Hortscience; 2008 43:1377–1381. 10.5194/amt-3-177-2010

[67] Machemer K, Shaiman O, Salts Y, Shabtai S, Sobolev I, Belausov E, et al Interplay of MYB factors in differential cell expansion, and consequences for tomato fruit development. Plant J; 2011 68:337–350. 10.1111/j.1365-313x.2011.04690.x 21707804

[68] Li T, Tan D, Liu Z, Jiang Z, Wei Y, Zhang L, Li X, Yuan H, Wang A. Apple MdACS6 Regulates Ethylene Biosynthesis During Fruit Development Involving Ethylene-Responsive Factor. Plant Cell Physiol; 2015 56:1909–1917. 10.1093/pcp/pcv111 26209510

[69] Liu X, Wang T, Bartholomew E, Black K, Dong M, Zhang Y, et al Comprehensive analysis of NAC transcription factors and their expression during fruit spine development in cucumber (*Cucumis sativus* L.). Hortic Res; 2018 5:31 10.1038/s41438-018-0036-z 29872536 PMC5981648

[70] Moyano E, Martínez-Rivas FJ, Blanco-Portales R, Molina-Hidalgo FJ, Ric-Varas P, Matas-Arroyo AJ, et al Genome-wide analysis of the NAC transcription factor family and their expression during the development and ripening of the *Fragaria* × *ananassa* fruits. PLoS One; 2018 13:e0196953 10.1371/journal.pone.0196953 29723301 PMC5933797

[71] Cheng Y, Ahammed GJ, Yu J, Yao Z, Ruan M, Ye Q, et al Putative WRKYs associated with regulation of fruit ripening revealed by detailed expression analysis of the WRKY gene family in pepper. Sci Rep; 2016 6:39000 10.1038/srep39000 27991526 PMC5171846

