# Supplementary material for: High-density genetic linkage-map construction of hawthorn and QTL mapping for important fruit traits
Source: PLoS One. 2020 Feb 11;15(2):e0229020. doi: 10.1371/journal.pone.0229020 (PMC7012432; doi:10.1371/journal.pone.0229020)
Supplement: S1 Fig — (PDF) [file pone.0229020.s001.pdf]

High-density genetic linkage-map construction of hawthorn and QTL mapping for important fruit traits

Yuhui Zhao[1], Yidi Zhao[1], Yinshan Guo [1,3*], Kai Su[1], Xiaochang Shi[1], Di Liu [1], Jijun Zhang[2*]

[1]College of Horticulture, Shenyang Agricultural University, Shenyang, P.R.C.

[2]College of Horticulture Science and Technology, Hebei Normal University of Science and technology, Qinhuangdao, P.R.C.

[3]National and Local Joint Engineering Research Center of Northern Horticultural Facilities Design and Application Technology, Shenyang, P.R.C.

[*]Corresponding author. Email: guoyinshan77@126.com, zjjqhd@163.com

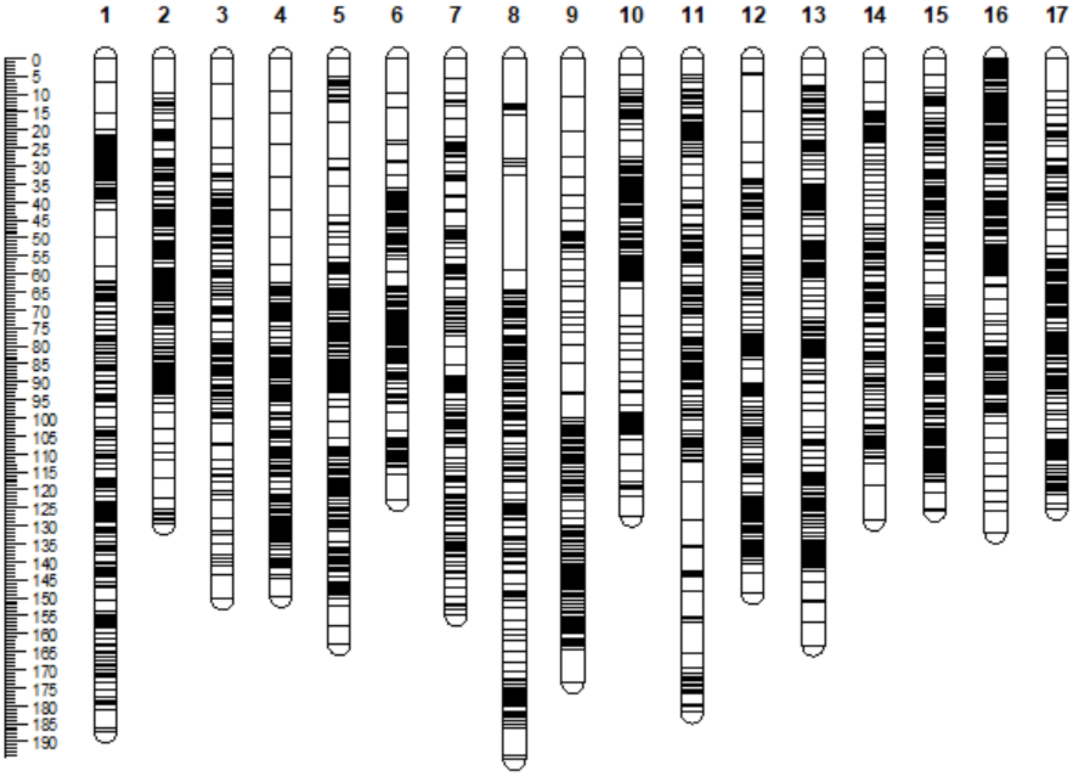
1
2
3
4
5
6
7
8
9
10
11
12
13
14
15
16
17
0
5
10
15
20
25
30
35
40
45
50
55
60
65
70
75
80
85
90
95
100
105
110
115
120
125
130
135
140
145
150
155
160
165
170
175
180
185
190
